# Patient expectations and treatment outcomes in dentistry: A questionnaire-based study

**DOI:** 10.6026/973206300222771

**Published:** 2026-05-31

**Authors:** Qalbi Fatima, Pallavi Priya, Imran Md, Vaibhava Raaj, Subhash Kumar, Abhishek Sinha

**Affiliations:** 1 Department of Dentistry, Patna Medical College & Hospital, Patna, Bihar, India

**Keywords:** Dental treatment, government hospital, patient satisfaction, patient expectations, treatment outcomes

## Abstract

Mismatch between patient expectations and perceived treatment outcomes remain a significant challenge affecting satisfaction and service utilization in government dental healthcare settings. Therefore, it is of interest to evaluate patient expectations before treatment and corresponding perceived outcomes after treatment at a government dental facility. A cross-sectional questionnaire-based study was conducted among 400 patients using validated Likert-scale instruments assessing domains such as pain management, functional restoration, esthetics, communication and facility experience. While pain management showed the highest expectation and satisfactory outcomes, significant gaps were observed in facility experience and waiting time, with only 43.5% of patients reporting outcomes meeting or exceeding expectations. Thus, we show the need to improve non-clinical service aspects alongside clinical care to enhance overall patient satisfaction and healthcare delivery.

## Background:

The idea of patient-centered care has become one of the key paradigms of modern healthcare provision where the treatment success measured by provider-related measures are replaced by the introduction of patient opinions, preferences and personal experiences as the primary factors determining the quality of healthcare [1]. In the context of this patient-centered approach, the evaluation of patient expectations and the extent to which achieved treatment outcomes meet them have been identified as an important dimension of healthcare quality assessment that determines not only patient satisfaction but also treatment adherence, clinical outcomes and reputation of the institution [2]. The concept of expectation-outcome relationship is especially relevant in the field of dental healthcare when making treatment decisions typically require complex tradeoffs of functional, esthetic, comfort and economic factors that are highly subjective and highly individual. Patient expectations are the expectations of the expected quality of care, treatment outcomes and experiences of services that individuals hold at the beginning of their healthcare interactions based on a complicated interconnection of past experiences, cultural assumptions, social forces, media and peer sources of information and the perceived attributes of the health care facility and health care professionals [3]. These expectations act as the cognitive barometers upon which patients then compare their real treatment experience situations with and thus they serve as potent predictors of perceived satisfaction irrespective of the objective clinical quality of care provided. The theory, which was initially formulated in the context of consumer behavior and later applied to healthcare, is the expectation-confirmation theory, which states that, when perceived outcomes are as high as or higher than expectations, a person develops satisfaction, whereas, when perceived outcomes are lower than expected, a person is satisfied with disappointment[4]. Patient expectations in dental healthcare setting are multidimensional in that they involve clinical, functional and esthetic improvement outcomes, as well as process related expectations such as waiting times, quality of communication, adequacy of explanation of the treatment, cleanliness of the facility, modernity of equipment and politeness of the staff [5]. Relative significance of these various expectation domains will differ in patients of different populations and will depend upon demographic factors, culture, economic status and the nature of presenting dental complaint. Knowledge of the hierarchical nature of patient expectations - which areas are of the greatest concern and where they are most vulnerable to negative disconfirmation - is something useful to work on in specific quality improvement programs [6].

Government dental centers in India have a special role in the example of healthcare, as the main point of access to affordable dental services by millions of poor people and have a vast amount of resources that can restrict their ability to serve all patients with their expectations [7]. Such facilities are typically characterized by high-quality clinical care performed by qualified people who may also involve special services at little to no charge to the patient. Nevertheless, they are often confronted with difficult issues in terms of high patient flow; long wait lists, inadequate physical facility, equipment upkeep and less staff to patient's proportions than those in the private sector [8]. The patient experience would be influenced by the quality of clinical competency of care received as well as the nature of the operational environment of the service delivery space. Other research studies exploring patient satisfaction in government dental facilities in India have largely found moderate levels of patient satisfaction, although clinical aspects of the care received higher ratings than administrative and environmental ones [9]. A majority of these studies, however, have measured satisfaction as a post-hoc measure without the systematic measurement of the pre- treatment expectations, thus they have not systematically examined the expectation-outcome comparison which gives a better understanding of the origin and the degree of satisfaction or dissatisfaction. In the absence of the knowledge of what the patients anticipated in the treatment, one cannot establish whether the reported dissatisfaction is attributed to objectively low results or they were based on unrealistically high expectations that no possible service provision model could meet [10]. The limited investigations that have utilized the expectation-outcome comparison techniqueologies in the dental practice context have been largely undertaken in the Western healthcare setting or even in the private practice environments where the dynamics of expectations may not be the same as those experienced within the setting of government hospitals in developing countries [11]. Individuals visiting governmental institutions could have varying levels of expectations in their baseline due to their knowledge of resource limitations, prior experiences in governmental institutions and culturally particular visions of what is acceptable in the form of governmental healthcare. This limits the applicability of the results in the Western or private-sector to the situation in the government dental facility in Bihar. The state of Bihar is an especially relevant scenario in the context of this investigation because it has a unique patient population in the state. The patients visiting Government Medical College and Hospital, Patna, are of varied socioeconomic status, education and geographic origin with a large number of them having to travel long distances through rural and semi-urban centres to seek dental specialty services which they find unavailable at the primary health care centres [12]. Therefore, it is of interest to evaluate the discrepancy between patient expectations and perceived treatment outcomes in dental care at Government Medical College and Hospital, Patna.

## Materials and Methods:

## Study design and setting:

This prospective cross-sectional questionnaire-based study was conducted at the Department of Dentistry, Government Medical College and Hospital (GMCH), Patna, Bihar, India, over a period of Seven months from September 2025 to March 2026. The dental department at GMCH, Patna provides comprehensive dental services through multiple specialty divisions including oral medicine and radiology, conservative dentistry and endodontics, prosthodontics, periodontics, oral and maxillofacial surgery, orthodontics and Pedodontics, serving an average daily outpatient volume of approximately 80 to 100 patients. Written informed consent was obtained from all participants in Hindi after thorough explanation of the study purpose, procedures, and voluntary nature of participation and assurance of data confidentiality.

## Sample size calculation:

Sample size was calculated using the formula n = Z^2^pq/d^2^,where Z = 1.96 (95% confidence level), p = estimated proportion of patients with met expectations (assumed 45% based on available literature from similar settings),q = 0.55 and d = margin of error (5%).The minimum sample was 381 patients. Accounting for an anticipated 15% loss to follow-up between the pre-treatment and post-treatment assessment, 470 patients were initially enrolled. Of these, 400 completed both the pre-treatment and post-treatment questionnaires and constituted the final study sample.

## Participant selection:

Patients attending the dental OPD who were scheduled to undergo at least one definitive dental treatment procedure were consecutively recruited.

## Inclusion criteria:

[1] Age 18 years and above

[2] Scheduled to undergo dental treatment at the facility (extraction, restoration, scaling, root canal treatment, prosthetic rehabilitation, or minor oral surgical procedure)

[3] Willing to return for post-treatment assessment or available for telephone follow-up

[4] Ability to comprehend the questionnaire in Hindi or English

[5] Provision of written informed consent

## Exclusion criteria:

[1] Patients presenting exclusively for consultation or radiographic investigation without treatment

[2] Patients with emergency conditions requiring immediate intervention precluding pre-treatment questionnaire administration

[3] Patients with cognitive impairment or communication barriers

[4] Patients undergoing long-term treatment extending beyond three months (orthodontics, complex implant rehabilitation)

[5] Healthcare professionals or dental students seeking treatment

## Study instrument development:

A paired questionnaire instrument was developed, comprising a Pre-Treatment Expectation Questionnaire (PTEQ) and a Post-Treatment Outcome Questionnaire (PTOQ). The instruments were designed to measure expectations and perceived outcomes across five parallel domains using identical dimensional frameworks.

## Instrument development process:

[1] Literature review and item generation: A comprehensive review of patient expectation and satisfaction instruments in dental literature was conducted. Preliminary items were generated through expert consultation and patient focus group discussions (two sessions, 8 patients each) to identify locally relevant expectation and outcome themes.

[2] Expert validation: A panel of five experts (two senior dental faculty, one health services researcher, one psychometrician and one hospital administrator) reviewed the items for content validity, clarity and cultural appropriateness. Content validity index was 0.89.

[3] Translation: Instruments were developed in English, translated to Hindi by two bilingual translators and back-translated to verify equivalence.

[4] Pilot testing: Both instruments were pilot tested on 40 patients (excluded from the main study). Cronbach's alpha was 0.86 for the PTEQ and 0.88 for the PTOQ. Test-retest reliability (two-week interval, n = 20) yielded an intraclass correlation coefficient of 0.84.

## Instrument structure:

Pre-treatment expectation questionnaire (PTEQ) - 25 items across five domains:

[1] Domain 1 - Pain management expectations (5 items): Expected pain relief, anticipated procedural pain control , expected effectiveness of anesthesia, expected post-operative pain management, expected speed of symptom resolution.

[2] Domain 2 - Functional restoration expectations (5 items): Expected improvement in chewing ability, speech improvement, restoration of tooth structure, durability of treatment outcome, overall functional improvement.

[3] Domain 3 - Esthetic improvement expectations (5 items): Expected improvement in dental appearance, color matching of restorations, smile enhancement, overall facial appearance improvement, expected cosmetic satisfaction.

[4] Domain 4 - Communication and information expectations (5 items): Expected treatment explanation adequacy, expected involvement in treatment decisions, expected information about alternatives, expected clarity regarding cost and duration, expected postoperative instruction provision.

[5] Domain 5 - Facility experience expectations (5 items): Expected cleanliness and hygiene, expected waiting time acceptability, expected staff courtesy, expected equipment quality, expected overall comfort.

Each item was scored on a five-point Likert scale (1 = very low expectation, 5 = very high expectation). Domain scores were calculated as mean item scores per domain (range 1-5). Overall expectation score was the mean of all domain scores.

## Post-treatment outcome questionnaire (PTOQ) - 25 parallel items:

Identical structure and domains as the PTEQ, but worded to assess perceived actual outcomes following treatment (1 = very poor outcome, 5 = very good outcome).

## Expectation-outcome discrepancy (EOD) scores:

Calculated as: EOD = Outcome Score - Expectation Score. Positive EOD indicates expectations exceeded (positive disconfirmation), zero indicates expectations met and negative EOD indicates expectations unmet (negative disconfirmation).

## Data collection procedure:

[1] Phase 1 - Pre-treatment assessment: The PTEQ was administered to enrolled patients in the OPD waiting area prior to their treatment appointment by trained research assistants through face-to-face interview. Socio-demographic data and treatment-related information (type of treatment scheduled, previous dental experience, referral source) were simultaneously collected.

[2] Phase 2 - Post-treatment assessment: The PTOQ was administered to the same patients within 7 to 14 days following completion of their dental treatment. For patients attending follow-up appointments, the questionnaire was administered in person at the facility. For patients who did not return within two weeks, telephone-based administration was conducted using a standardized protocol. The treatment type actually received and numbers of visits required were documented.

## Statistical analysis:

Data were analyzed using SPSS version 26.0 (IBM Corp., Armonk, NY, USA). Descriptive statistics were expressed as mean ± standard deviation, frequencies and percentages. Paired t-tests were used to compare pre-treatment expectation and post-treatment outcome scores within domains. Independent samples t-tests and one-way ANOVA with post-hoc Tukey's test were used for between-group comparisons. Wilcoxon signed-rank tests were used where data distribution deviated from normality. Pearson's correlation coefficients assessed relationships between continuous variables. Multiple linear regression analysis was performed with overall EOD score as the dependent variable. Effect sizes were calculated using Cohen's d. Statistical significance was set at p < 0.05.

## Results:

A total of 400 participants were included in the study. The mean age was 35.4 ± 11.8 years, with an age range of 18-68 years. Males constituted 218 participants (54.5%). Most participants had secondary school education or above, while 51 participants (12.8%) were illiterate. Urban residents constituted 36.0%, followed by rural residents (32.5%) and semi-urban residents (31.5%). The most common treatment performed was extraction (31.0%), followed by restoration/filling (22.5%), root canal treatment (15.0%), scaling and polishing (14.5%), prosthetic rehabilitation (10.5%) and minor oral surgery (6.5%). First-time dental visitors accounted for 99 participants (24.8%). The comparison of pre-treatment expectation scores and post-treatment outcome scores across different domains is shown in Table 1. All domains demonstrated statistically significant negative expectation-outcome discrepancy (EOD) scores, indicating that perceived outcomes were lower than initial expectations. The smallest discrepancy was observed for pain management (-0.24 ±0.68), suggesting relatively better expectation fulfillment in this domain. The largest discrepancy was observed for facility experience (-0.58 ± 0.92), followed by communication and information (-0.46 ± 0.78), esthetic improvement (-0.44 ± 0.84), and functional restoration (-0.42 ± 0.74). The overall expectation score was 4.00 ±0.58, while the overall outcome score was 3.58 ± 0.68, giving a significant overall EOD score of -0.42 ± 0.64 (p < 0.001). Expectation fulfillment categories are presented in Table 2 Overall, expectations were exceeded in 68 participants (17.0%) and met in 106 participants (26.5%). Thus, 174 participants (43.5%) reported that their expectations were either met or exceeded. Mild negative discrepancy was observed in 134 participants (33.5%), moderate negative discrepancy in 64 participants (16.0%) and severe negative discrepancy in 28 participants (7.0%). Treatment-specific analysis showed that prosthetic rehabilitation had the highest negative mean EOD score (-0.62 ± 0.78), followed by root canal treatment (-0.52± 0.71), minor oral surgery (-0.48 ±0.68), restoration/filling (-0.38 ± 0.62), scaling and polishing (-0.34 ± 0.56), and extraction (-0.31 ± 0.58). Post-hoc Tukey analysis showed that prosthetic rehabilitation had significantly greater negative discrepancy compared with extraction (p = 0.004) and scaling and polishing (p = 0.012). Domain-specific fulfillment analysis showed that pain management had the highest proportion of expectations met or exceeded (62.8%), followed by functional restoration (46.2%), esthetic improvement (41.5%), communication and information (39.8%), and facility experience (31.0%). Factors associated with overall EOD are summarized in Table 3. A significant association was observed between education level and EOD score (p = 0.002), with greater negative discrepancy among participants with graduate-level education or above (-0.56 ± 0.72). Residential background was also significantly associated with EOD (p = 0.008), with urban participants showing greater negative discrepancy (-0.52 ± 0.68) compared with semi-urban (-0.41 ± 0.62) and rural participants (-0.32 ± 0.58). Participants with previous dental experience had a greater negative EOD score (-0.46 ± 0.68) than first-time visitors (-0.34 ± 0.56; p = 0.036). Waiting time showed a significant graded association with EOD, with the most negative discrepancy observed among patients waiting more than 2 hours (-0.64 ± 0.78; p <0.001). Correlation analysis showed that overall EOD had significant negative correlations with education level (r = -0.28, p <0.001), pre-treatment expectation score (r = -0.54, p <0.001), number of visits required (r = -0.32, p <0.001), and waiting time (r = -0.46, p <0.001). Age showed a weak positive correlation with EOD (r = 0.12, p = 0.016). Multiple regression analysis identified pre-treatment expectation score as the strongest independent predictor of negative EOD (β = -0.38, p <0.001), followed by waiting time (β = -0.26, p <0.001), number of visits required (β = -0.17, p = 0.002), education level (β = -0.14, p = 0.008), prosthetic treatment type (β = -0.12, p = 0.018), and perceived communication adequacy (β = 0.21, p <0.001). Previous dental experience and age were not significant independent predictors. The regression model was statistically significant and explained 52% of the variance in overall EOD score (R ^2^ = 0.52, F = 52.87, p <0.001).

## Discussion:

The current research presents the first serious assessment of the expectation-outcome correlation among dental patients in a government medical college hospital in Bihar and a subtle image where the rate of clinical treatment results essentially meets patient anticipations and service delivery and facility experience are grossly inadequate. The result that 43.5% of patients stated that they had their expectations met or surpassed and 56.5% reported having different levels of negative discrepancy shows that most patients walk out of the facility with some amount of unmet expectations, which has great implications to patient retention, treatment adherence and reputation. The domain analysis shows that there is apparent hierarchy of expectation achievement that depicts inherent strengths and weaknesses of government dental facilities. The area of pain management showed the least expectation-outcome disparity and the largest fulfillment percentage (62.8), meaning that the major clinical role of dental treatment, which is the mitigation of pain and management of dental emergencies, is being fulfilled with reasonable efficiency. This result is encouraging and is in line with the clinical competency that defines the government medical college departments where the treatment is administered by qualified faculty and under the supervision of trainees with access to standard pharmacological and procedural protocols of managing pain [13]. The increasingly greater negative differences between the actual and expected values in the functional restoration, esthetic improvement, and communication and facility experience domains indicate the spheres, in which the service delivery enhancement is most urgently required. The domain with the greatest gap (-0.58) was the facility experience one and the major issues that contributed to the gap were waiting time dissatisfaction, perceived inadequacy of infrastructure and environmental comfort issues. Waiting time has also been reported to be one of the most important causes of patient dissatisfaction in the government healthcare facilities in the developing nations, where large numbers of patients compared to their services available create long waiting times that entail high opportunity costs to the patient in terms of lost wages, traveling inconvenience and care-giver activities [14]. The fact that waiting time management is a high-priority patient experience goal is proven by the fact that waiting time is significantly negatively related to overall EOD score (r = -0.46) and emerges as the second strongest predictor in the regression model. It is theoretically justified that pre-treatment expectation level appeared to be the most significant predictor of negative discrepancy (0.38) as it has been previously found in the research of healthcare satisfaction in numerous medical specialties. When patients have higher expectations at the beginning, the cognitive benchmark on which they base their results is higher and it is therefore more challenging to get the service experience to match their expectations [15]. The relationship was also evidenced by the fact that patients with higher education and residing in urban areas, who presumably have more exposure to the services of a private dentist and the media-mediated images of the modern dental practice, showed higher negative EOD scores than rural patients and patients with lower educational levels, whose expectations were also more humble and thus more easily fulfilled. The treatment-specialized analysis has indicated that the highest scores of negative discrepancies were produced by the prosthetic rehabilitation and this fact can be attributed to the multifactorial and multifactorial character of the treatment expectations of the prosthetic treatment. This is because patients needing denture or crowns usually have expectations concerning comfort, natural appearance and instant functional restoration that might not be entirely achievable especially with the traditional materials and methods of making prosthetic available at the government hospitals [16].

## Discussion:

The current research presents the first serious assessment of the expectation-outcome correlation among dental patients in a government medical college hospital in Bihar and a subtle image where the rate of clinical treatment results essentially meets patient anticipations and service delivery and facility experience are grossly inadequate. The result that 43.5% of patients stated that they had their expectations met or surpassed and 56.5% reported having different levels of negative discrepancy shows that most patients walk out of the facility with some amount of unmet expectations, which has great implications to patient retention, treatment adherence and reputation. The domain analysis shows that there is apparent hierarchy of expectation achievement that depicts inherent strengths and weaknesses of government dental facilities. The area of pain management showed the least expectation-outcome disparity and the largest fulfillment percentage (62.8), meaning that the major clinical role of dental treatment, which is the mitigation of pain and management of dental emergencies, is being fulfilled with reasonable efficiency. This result is encouraging and is in line with the clinical competency that defines the government medical college departments where the treatment is administered by qualified faculty and under the supervision of trainees with access to standard pharmacological and procedural protocols of managing pain [13]. The increasingly greater negative differences between the actual and expected values in the functional restoration, esthetic improvement, and communication and facility experience domains indicate the spheres, in which the service delivery enhancement is most urgently required. The domain with the greatest gap (-0.58) was the facility experience one and the major issues that contributed to the gap were waiting time dissatisfaction, perceived inadequacy of infrastructure and environmental comfort issues. Waiting time has also been reported to be one of the most important causes of patient dissatisfaction in the government healthcare facilities in the developing nations, where large numbers of patients compared to their services available create long waiting times that entail high opportunity costs to the patient in terms of lost wages, traveling inconvenience and care-giver activities [14]. The fact that waiting time management is a high-priority patient experience goal is proven by the fact that waiting time is significantly negatively related to overall EOD score (r = -0.46) and emerges as the second strongest predictor in the regression model. It is theoretically justified that pre-treatment expectation level appeared to be the most significant predictor of negative discrepancy (0.38) as it has been previously found in the research of healthcare satisfaction in numerous medical specialties. When patients have higher expectations at the beginning, the cognitive benchmark on which they base their results is higher and it is therefore more challenging to get the service experience to match their expectations [15]. The relationship was also evidenced by the fact that patients with higher education and residing in urban areas, who presumably have more exposure to the services of a private dentist and the media-mediated images of the modern dental practice, showed higher negative EOD scores than rural patients and patients with lower educational levels, whose expectations were also more humble and thus more easily fulfilled. The treatment-specialized analysis has indicated that the highest scores of negative discrepancies were produced by the prosthetic rehabilitation and this fact can be attributed to the multifactorial and multifactorial character of the treatment expectations of the prosthetic treatment. This is because patients needing denture or crowns usually have expectations concerning comfort, natural appearance and instant functional restoration that might not be entirely achievable especially with the traditional materials and methods of making prosthetic available at the government hospitals [16].

The numerous appointment obligations, long production schedules and accommodation phase involved in the treatment of a prosthetic also adds to expectation-outcome discrepancies. On the other hand, extraction and scaling showed significantly fewer discrepancies, probably because of the procedurally simple nature and instantly noticeable consequences of these interventions. The strong positive relationship between the perceived communication adequacy and the EOD score reflects the potent moderating role of effective communication between the providers and patients on treatment satisfaction. Detailed pre-treatment explanation that sets realistic expectations, the details of the procedures, possible limitations and addressing the patients concerns have been demonstrated to establish expectations to realistic levels and improve perceived outcome quality despite objective outcomes being the same [17]. The practical implication of this discovery is relevant in the direct clinical practice in government dental departments where time limited consultations have the effect of omitting certain elements of patient education. It is interesting that the observation of a smaller negative EOD by first-time dental visitors than by patients with prior dental experience is also interesting and could be due to an expectancy-calibration effect. Patients who have not been exposed to dental care before might still have fairly unformed expectations t hat can more easily be fulfilled by the reality of their service experience, but patients who have previous dental experience, especially at the privately available facilities, will bring in standards of comparison (with alternative healthcare interactions) which might not be met by the government facilities [18]. This result indicates that initial dental visits are important occasions of creating a favorable institutional image to influence subsequent expectations and service commitment. The number of visits to complete treatment proved to be a major negative predictor of the EOD score, showing the cumulative burden that the many visits have on patients visiting the government facilities. Any additional visit implies a repeated waiting time, traveling, lost time at work and re-exposure to the institutional setting, increasing the negative experience [19]. The reduction in the number of visits by providing better appointment scheduling, single-visit treatments where clinically suitable and effective sequencing of treatment would go a long way in improving the patient experience. The moderate overall R2 value of 0.52 of the regression model shows that the measured factors play a role in generating about a half of the variance of expectation-outcome discrepancy which means that there are other unmeasured factors (such as individual psychological characteristics, provider-specific interpersonal skills, treatment specific clinical outcomes and the role serving family members) that play a role in the expectation-outcome relationship and should be studied in future research [20]. The domain of esthetic improvement showed a significant gap (mean EOD = -0.44) that deserves being paid particular attention to. The subjectivity of esthetic expectations in dentistry is a persistent aspect and depends on the representations of ideal smiles in the media, the culture of celebrity culture and the salience of cosmetic dental marketing, which can determine aspirational norms that are beyond the reach of government-provided facilities that prioritize functional rehabilitation of the mouth [21]. This gap could be alleviated with the help of realistic counseling, visual interventions on the expected results and sincere discussion of the material and technical constraints that exist at government facilities. There are a number of limitations that should be mentioned. The fact that consecutive sampling was done at one institution can be a limitation to generalizability. The self-reported perceptions in both the pre-treatment and post-treatment assessment did not involve objective clinical outcome measure and instead, subjectivity. The 7-14 days post-treatment period is when short-term perceived outcomes are recorded and maybe not long-term satisfaction especially in the case of prosthetic where the adaptation takes place during a period of weeks to months. Follow-up on non-returning patients using telephone can produce response patterns that are not directly related to face-to-face evaluation. In the face-to-face interview type, social desirability bias might affect the responses [22]. The evidence base would be enhanced by future longitudinal research using several follow-ups, objective outcome measures and qualitative investigation of how expectation is formed.

## Conclusion:

We show that while clinical outcomes, particularly pain management, largely meet patient expectations, significant gaps persist between expectations and perceived outcomes in non-clinical domains such as facility experience and communication. Higher expectations, prolonged waiting time and complex treatment needs are associated with greater dissatisfaction, emphasizing the role of expectation management. Improving communication, reducing waiting time and enhancing infrastructure are essential to bridge expectation-outcome gaps and improve overall patient satisfaction in government dental settings.

## Figures and Tables

**Table 1:** Pre-treatment expectation scores, post-treatment outcome scores and expectation-outcome discrepancy by domain (n = 400)

**Domain**	**Expectation Score Mean ± SD**	**Outcome Score Mean ±SD**	**EOD Score Mean ± SD**	**Cohen's d**	**p-value (Paired t-test)**
Pain Management	4.32 ± 0.61	4.08 ± 0.72	-0.24 ± 0.68	0.36	< 0.001
Functional Restoration	4.18 ± 0.67	3.76 ± 0.78	-0.42 ± 0.74	0.58	< 0.001
Esthetic Improvement	3.86 ± 0.82	3.42 ± 0.86	-0.44 ± 0.84	0.52	< 0.001
Communication & Information	3.94 ± 0.73	3.48 ± 0.81	-0.46 ± 0.78	0.6	< 0.001
Facility Experience	3.72 ± 0.79	3.14 ± 0.87	-0.58 ± 0.92	0.7	< 0.001
Overall Score	4.00 ± 0.58	3.58 ± 0.68	-0.42 ± 0.64	0.67	< 0.001

**Table 2:** Expectation fulfillment categories and treatment-specific outcomes

**Panel A: Overall Expectation Fulfillment**	**n (%)**
Expectations exceeded (EOD > 0)	68 (17.0%)
Expectations met (EOD = 0, ± 0.25)	106 (26.5%)
Mild negative discrepancy (EOD -0.26 to -0.75)	134 (33.5%)
Moderate negative discrepancy (EOD -0.76 to -1.25)	64 (16.0%)
Severe negative discrepancy (EOD < -1.25)	28 (7.0%)
Total with expectations met or exceeded	174 (43.5%)
Panel B: Mean EOD by Treatment Type	Mean EOD ± SD
Extraction	-0.31 ± 0.58
Restoration/filling	-0.38 ± 0.62
Root canal treatment	-0.52 ± 0.71
Scaling and polishing	-0.34 ± 0.56
Prosthetic rehabilitation	-0.62 ± 0.78
Minor oral surgery	-0.48 ± 0.68
Panel C: Domain-Specific Fulfillment (% Expectations Met or Exceeded)	
Pain management	62.80%
Functional restoration	46.20%
Esthetic improvement	41.50%
Communication & information	39.80%
Facility experience	31.00%

**Table 3:** Factors associated with overall expectation-outcome discrepancy score

**Panel A: EOD by sociodemographic factors**	**Mean EOD ± SD**	**p-value**
**Education Level**		
Illiterate	-0.26 ± 0.52	
Primary school	-0.34 ± 0.58	
Secondary school	-0.42 ± 0.64	0.002 (ANOVA)
Higher secondary	-0.48 ± 0.67	
Graduate and above	-0.56 ± 0.72	
**Residential Background**		
Urban	-0.52 ± 0.68	
Semi-urban	-0.41 ± 0.62	0.008 (ANOVA)
Rural	-0.32 ± 0.58	
**Previous Dental Experience**		
First-time visitor	-0.34 ± 0.56	0.036 (t-test)
Previous dental experience	-0.46 ± 0.68	
**Waiting Time Experienced**		
< 1 hour	-0.28 ± 0.54	
1-2 hours	-0.42 ± 0.62	< 0.001 (ANOVA)
> 2 hours	-0.64 ± 0.78	
**Panel B: Correlations with Overall EOD**	**r**	**p-value**
Age	0.12	0.016
Education level	-0.28	< 0.001
Pre-treatment expectation score	-0.54	< 0.001
Number of visits required	-0.32	< 0.001
Waiting time	-0.46	< 0.001
**Panel C: Multiple Regression - DV: Overall EOD**	**β**	**p-value**
Pre-treatment expectation score	-0.38	< 0.001
Waiting time experienced	-0.26	< 0.001
Number of visits required	-0.17	0.002
Education level	-0.14	0.008
Communication adequacy (perceived)	0.21	< 0.001
Treatment type (prosthetic = 1)	-0.12	0.018
Previous dental experience	-0.08	0.124
Age	0.06	0.236
R _2_= 0.52, F = 52.87, p < 0.001		
